# Facebook Use and Perceived Social Support Among Older Adults Who Live Alone

**DOI:** 10.1093/geroni/igaf122.1347

**Published:** 2025-12-31

**Authors:** Kristine Ajrouch, Jess Francis-Levin

**Affiliations:** University of Michigan, Ann Arbor, Michigan, United States; University of Michigan, ANN ARBOR, Michigan, United States

## Abstract

Older adults (aged 65+) living alone are at especially high risk of experiencing social isolation. Prior research has shown that Facebook communication is associated with social connectedness, serving as a protective resource against the negative influences of social isolation. Yet, Facebook communication is complex, offering various domains of engagement. This study will assess the impact of three separate of Facebook communication domains (broadcasting, directed, consumption) and their association with perceived social support among older adults who live alone. Data were collected through an online survey of older Facebook users who were living alone, unmarried, and without children (n = 517; M age=70). Participants were asked to respond to survey questions related to social support, Facebook use, and socio-demographics. We also asked participants to provide responses to an open-ended item inquiring about the role of Facebook in their daily life. Approximately 50% were women who reported on average some level of college education. Multiple regression analysis showed a positive association between the frequency of Facebook communication and perceptions of social support (β =.88; p = .001). Specifically, our findings indicate that the frequency of directed communication has a significant and positive association with social support (β =.22; p = .01). There were no significant associations between frequency of broadcasting communication or consumption communication with social support. Findings will be discussed through the lens of social relations theories to elaborate the benefits and barriers of Facebook use for facilitating perceived social support among older adults who live alone.

